# Variable neutralizing antibody responses to 10 SARS-CoV-2 variants in natural infection with wild- type (B.1) virus, Kappa (B.1.617.1), and Delta (B.1.617.2) variants and COVISHIELD vaccine immunization in India: utility of the MSD platform

**DOI:** 10.3389/fimmu.2023.1181991

**Published:** 2023-06-05

**Authors:** Rajashree Patil, Sonali Palkar, Akhileshchandra Mishra, Rahul Patil, Vidya Arankalle

**Affiliations:** ^1^ Department of Communicable Diseases, Interactive Research School for Health Affairs, Bharati Vidyapeeth (Deemed to be) University, Pune, Maharashtra, India; ^2^ Department of Pediatrics, Bharati Vidyapeeth Medical College, Bharati Vidyapeeth (Deemed to be) University, Pune, Maharashtra, India

**Keywords:** COVISHIELD, MSD, SARS-COV-2 variants, immunogenicity, ACE2 neutralization

## Abstract

For the efficacy of COVID-19 vaccines, emergence of variants accumulating immune-escape mutations remains a major concern. We analyzed the anti-variant (n = 10) neutralization activity of sera from COVID-19 patients infected with Wuhan (B.1), Kappa, and Delta variants and COVISHIELD vaccine recipients with (prepositives) or without (prenegatives) prior antibody positivity using V- PLEX ACE2 Neutralization Kit from MSD. MSD and PRNT_50_ correlated well (r = 0.76–0.83, p < 0.0001). Despite the least antibody positivity in Kappa patients, anti-variant neutralizing antibody (Nab) levels in the responders were comparable with Delta patients. Vaccinees sampled at 1 month (PD2-1) and 6 months (PD2-6) post-second dose showed the highest seropositivity and Nab levels against the Wuhan strain. At PD2-1, the responder rate was variant-dependent and 100% respectively in prenegatives and prepositives. Nab levels against B.1.135.1, B.1.620, B.1.1.7+E484K (both groups), AY.2 (prenegatives), and B.1.618 (prepositives) were lower than that of Wuhan. At PD2-6, positivity decreased to 15.6%–68.8% in the prenegatives; 3.5%–10.7% of prepositives turned negative for the same four variants. As against the decline in Nab levels in 9/10 variants (prenegatives), a further reduction was seen against the same four variants in the prepositives. These variants possess immune-evasion-associated mutations in the RBD/S region. In conclusion, our data show that the Nab response of patients to multiple variants depends on the infecting variant. We confirm superiority of hybrid immunity in neutralizing multiple variants. Depending on the infecting variant pre- or postvaccination, immune response to different vaccines in different populations will vary and impact protection against emerging variants. The MSD platform provides an excellent alternative to live virus/pseudovirus neutralization tests.

## 1 Introduction

The COVID-19 pandemic is unique in the rapid development of vaccines employing conventional/novel platforms and global use. The emergence of SARS-CoV-2 variants of concern (VOCs) with increased transmissibility and altered antigenicity has been challenging in the effective control of the pandemic. D614G (B.1) was the first major mutation that was associated with increased transmissibility early in the pandemic (1, 2),. Subsequent differential evolution of the virus in different countries led to several variants with altered characteristics of public health importance. In 2020, Alpha (B.1.1.7), Beta (B.1.351), and Gamma (P.1) VOCs emerged respectively in the UK, South Africa, and Brazil (3–5). The circulation of these variants was limited to some geographic areas. Following initial detection in India in April 2021, the Delta (B.1.617.2) variant spread rapidly across the world (6). The Omicron (BA.1/BA.2) variants detected in Botswana and South Africa were transmitted globally in a short time span (7, 8). Additionally, several variants were reported without significant spread (B.1.1.318, Theta (C.36.3, P.3), Mu (B.1.621), B.1.620, B.1.617.3, Lambda (C.37), A.30, AT.1, B.1.638, and C.1.2) (9–14).

During clinical trials of COVID-19 vaccines, *in vitro* neutralization assays such as plaque reduction neutralization test (PRNT) and microneutralization test (MNT) were developed and used employing the Wuhan (wild type, lineage B) virus. These tests required a BSL-3 facility and hence could be performed by limited laboratories. Importantly, neutralizing antibodies (Nab) have been correlated with protection (15). Understanding the neutralization potential of vaccinees receiving different COVID-19 vaccines or patients infected with different variants against the VOCs remains a top priority. Since the circulation of variants and types of vaccines used for immunization vary in different countries, evaluation of immune responses in different populations is necessary. These results help policymakers to adopt suitable immunization policies. As vaccines were administered irrespective of the prior history of COVID-19, the immune response of the previously infected group was quickly determined (16). Even with one dose, prior infected individuals developed broader and higher neutralizing antibody responses (17, 18). As of 01/03/2023, the COVISHIELD vaccine contributed to 82.8% of 2.19 billion vaccine doses administered in India (geographicinsights.iq.harvard.edu/IndiaVaccine). The COVISHIELD™ vaccine is the complete equivalent of the AstraZeneca-ChAdOx1-S/nCoV-19 (recombinant) vaccine, a replication-deficient adenoviral vector vaccine developed by the University of Oxford (Oxford, UK).

For the use of PRNT/MNT in evaluating variant-specific Nab responses in the BSL-3 laboratory, all the emerging VOCs need to be generated/available, followed by standardization of tests with each variant. Although a pseudovirus neutralization test (PNT) can be performed in a BSL-2 lab, this approach requires various genetic manipulations and molecular expertise to construct variant-specific recombinant viruses. With commendable efforts, MNT with 14 variants has been reported (19). However, for most laboratories with limited molecular expertise, this may not be possible. Furthermore, even if the pseudoviruses are generated, the test requires mammalian tissue culture and a minimum of 24–48 h. With the availability of the Meso Scale Discovery (MSD) platform, an electrochemiluminescence-based (ECL) multiplex assay for the qualitative and quantitative assessment of neutralizing antibodies, we attempted to explore the utility of this assay in understanding variant-specific Nab responses of COVID-19 patients infected with different variants and COVISHIELD vaccine recipients with or without prior antibody positivity. The single-tube assay is quick (2.5 h) and can be performed in a BSL-2 laboratory. Unfortunately, at the time of conducting the current study, Omicron kits were under development and hence the study is restricted to non-Omicron VOCs and other variants.

## 2 Methodology

This study was approved by the institutional “Human Ethics Committee” (no. BVDUMC/IEC/185A).

### 2.1 Study population and clinical samples

The following groups were included in the study.

#### 2.1.1 IgG-SARS-CoV-2 negative controls

To determine the specificity of the MSD assay in relation to all the variants used, 38 plasma samples negative for IgG-anti-SARS-CoV-2 antibodies (ELISA) were used.

#### 2.1.2 COVID-19 patients

Serum/plasma samples from COVID-19 patients (n = 84) diagnosed by q-RT-PCR at Bharati Vidyapeeth (deemed to be) University Medical College and Hospital (BVDUMCH), a tertiary care hospital, were used. These included (1) 47 patients [M=27, F=20, median age-36 (24-54 years)] infected with the Wuhan-Hu-1 (wild-type, D614G) virus during the first wave (7 serum and 40 plasma samples) and (2) 18 patients [M=13, F=5, median age-45 (27-77 years)] infected with the B.1.617.1/Kappa lineage variant during the early part of the second wave and 19 patients [M=13, F=6, median age-41 (24-67 years)] infected subsequently with B.617.2/Delta lineage variant (all plasma samples). The variants were identified by sequencing the RBD/S gene (20).

#### 2.1.3 Recipients of COVISHIELD vaccine

The samples used for this study represent a subset of samples collected earlier for the evaluation of the immune response of healthcare personnel to COVID-19 vaccinees (21). Of these, samples with sufficient volumes (n=135) were selected for the study. From the naïve vaccinees, 43 [M=17, F=26, median age- 41 (21-71 years)] samples were collected at 1 month and 32 [M=12 and F=20, median age-42 (21-71 years)] samples were obtained at 6 months post-second dose. From vaccinees with prior exposure to the virus as evidenced by the presence of IgG-anti-SARS-CoV-2 antibodies (InBios International, Inc., USA), 32 [M=16 and F=16, median age-31 (23-54 years)] and 28 [M=14 and F=14, median age-35 (24-54 years)] samples were respectively collected at 1 and 6 months post-second dose.

Serum/plasma samples collected earlier from COVID-19 patients and vaccinees, tested for neutralizing antibody titers using PRNT_50_ and stored in aliquots at -70^0^C, were used.

### 2.2 Plaque reduction neutralization test

PRNT was done according to the method described previously (22). Briefly, Vero CCL81 cells were seeded in 24- well plates (1 × 10^5^ cells/ml) using MEM+10% FBS+100 I.U./ml penicillin and 100 (μg/ml) streptomycin medium, the day before the assay. 1:5 diluted serum samples were heat inactivated at 56°C for 30 min. Further fourfold serum dilutions were performed in MEM+2%FBS and mixed with 40–80 PFU/ml of SARS-CoV-2 virus (8004/IND/2020/IRSHA PUNE, accession number MT416726). This isolate (lineage B.1) exhibited a D614G mutation. Virus and serum samples were incubated for 1 h at 37°C in a humidified incubator with 5% CO_2_. After 1 h, 100 µl of the mixture was added in duplicates on Vero cells in 24- well plates, which were further incubated for 1h. One milliliter of 1% overlay media (MEM+Aquacide II (Merck) +2% FBS+100 I.U./ml penicillin and 100 (μg/ml) streptomycin) was added to the Vero cell monolayer. Cells were incubated for 5 days at 37°C in a humidified incubator with 5% CO_2_. After 5 days, cells were fixed with 3.7% formaldehyde, washed thrice with phosphate-buffered saline, and stained using 1% crystal violet. Plaques were counted using CTL ImmunoSpot SC 1.7.3.4001, and PRNT_50_ titer was calculated using the logistic regression method.

### 2.3 MSD ACE2 competition/neutralization assay

The assay was performed according to the instructions of the manufacturer. Precoated multiplex assay plates were provided in MSD V-PLEX neutralization kits (SARS CoV-2 panel-15, K15502U). There were 10 spike antigens of different SARS-CoV-2 variants (**Table 1**) coated in a single well of a 96-well plate. First, assay plates were blocked with 150 µl/well of MSD Blocker A solution at room temperature with shaking (~700–800 RPM) for 30 min. Serum samples were diluted 1:10 in MSD-Diluent 100, and the calibrator was diluted with the scheme provided by the manufacturer. Blocked plates were washed thrice with 150 µl of MSD wash buffer. Samples (25 µl) and calibrators (25 µl) were added in duplicates to the plate and incubated for 1 h at room temperature with shaking (~700–800 RPM). After 1 h, 1× SULFO-TAG human ACE2 protein solution (detection solution) (25 µl) was added to the same wells containing serum samples and plates were further incubated with shaking for 1 h. SULFO-TAG human ACE2 protein competes for precoated variants antigen with neutralizing antibodies present in serum samples. Plates were washed thrice with (150 µl per well) MSD wash buffer. Immediately after washing, MSD GOLD Read Buffer B (150 µl) containing the ECL substrate was added to the plate. Plates were read in the MESO QuickPlex SQ120 plate imager, where the current is applied to the plate, and if the coated antigen and ACE2 SULFO TAG have formed a complex, then in the presence of ECL substrate, it emits light, and MSD imager displays the output as the emitted light units. The light units were maximum for only diluent (without any serum sample) containing wells as all coated antigens can form a complex with ACE2 SULFO TAG and give maximum light units in the presence of the ECL substrate. Fold inhibition in light units is directly comparable to the number of neutralizing antibodies. Neutralizing antibodies compete with ACE2 SULFO TAG for viral antigen binding. Hence, lower light units correspond to higher neutralizing antibodies in samples. Fold inhibition was calculated by dividing diluents’ light units with test samples’ light units for matched spike antigens of variant. Values ≥1.5 are considered reactive for variant-specific antibodies.

**Table 1:** Variant-wise amino acid mutations in the Spike protein and impact on immunoreactivity in relation to Wuhan.

Variants	Common designation	Mutations* in the region of Spike	Significant reduction in Covishield-induced immunoreactivity in MSD assay			
			Prenegative		Prepositive	
			Y/N	Fold change **	Y/N	Fold change**
B.1	Wuhan-Hu-1	**D614G**	**-**	**-**	**-**	**-**
B.1.351.1	Botswana (Beta sub-lineage)	**K417N, E484K, N501Y,** D80A, D215G, **D614G,** A701V	YES	**2.22**	YES	**19.50**
B.1.620	Europe	**S477N, E484K**, P26S, **ΔH69-V70,** V126A, Δ**Y144**, Δ242-244, H245Y, **D614G, P681H,** T1027I, D1118H	YES	**2.16**	YES	**15.45**
AY.2	India (Delta sub-lineage)	**K417N, L452R, T478K**, T19R, V70F, G142D, E156G, Δ157/158, A222V, **D614G, P681R**, D950N	YES	**2.11**	NO	**-**
B.1.618	India	**E484K,** ΔY145/146, D614G	NO	**-**	YES	**5.66**
B.1.1.7+E484K	U.K. (Alpha sub-lineage)	**E484K, N501Y, ΔH69-V70, ΔY144,** A570D, **D614G, P681H**, T716I, S982A, D1118H	YES	**1.93**	YES	**20.88**
B.1.258.17	Europe	**N439K, ΔH69-V70,** L189F, **D614G,** V772I	NO	–	NO	–
B.1.617.2 +ΔY144	Vietnam (Delta sub-lineage)	**L452R, T478K,** T19R, **ΔY144**, Δ157/158, **D614G, P681R**, D950N	NO	–	NO	–
AY.1	Nepal (Delta sub-lineage)	**K417N, L452R, T478K,** T19R, T95I, G142D, E156G, Δ157/158, W258L, **D614G, P681R,** D950N	NO	–	NO	–
B.1.466.2	Indonesia	N439K, W152R, **D614G, P681R**	NO	–	NO	–

*Bold letters indicate mutations with confirmed immune evasion and enhanced transmissibility. **Fold change was calculated by dividing ACE2 competition values of the Wuhan virus with respective variants at PD2-1. Only significant fold changes are shown. '-' means no significant fold change.

### 2.4 Statistical analysis

GraphPad Prism 9.5.1 and R 4.1.3 were used for all analyses. For PRNT_50_ and MSD assay correlation analysis non-parametric Spearman correlation was used. ACE2 competition values were compared using the Kruskal–Wallis test followed by *post- hoc* Dunn’s test, as indicated in the respective figure legends. For the comparison between PD2-1, PD2-6, and prenegative and prepositive vaccinee groups, two-tailed Mann–Whitney- U test was performed.

## 3 Results

For understanding the neutralization potential of sera from infected and vaccinated individuals against multiple SARS-CoV-2 variants, we did not have access to live-virus or pseudovirus-based tests for such variants. With the availability of the MSD platform that can simultaneously detect and quantitate antibodies against multiple SARS-CoV-2 variants, we explored the use of the V-PLEX SARS-CoV-2 Panel 15 (ACE2) Kit available at the time of conducting this study. This is a multiplex assay for measuring the antibodies that block the binding of ACE2 to Spike antigens from variants of SARS-CoV-2 including the wild type-Wuhan, Alpha, Beta, and Delta variants and other variants present in the kit.

### 3.1 PRNT_50_ and MSD assays correlate well

**Figure 1:**
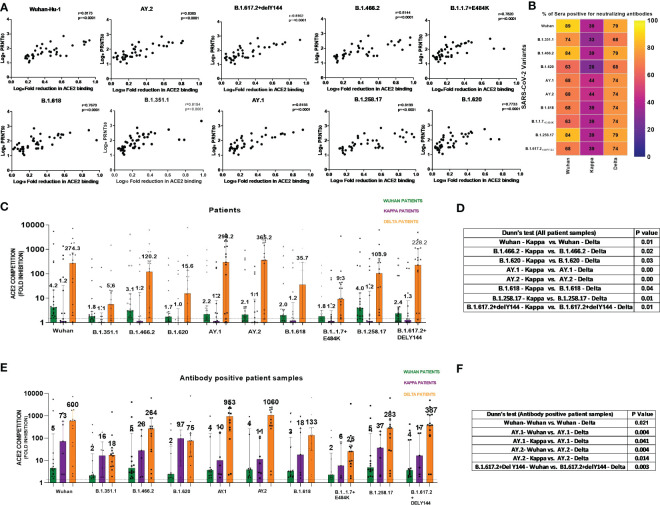
**(A)** For assessing the performance of MSD in relation to PRNT_50_ employing the Wuhan-Hu-1 strain, 47 samples (n = 7 serum, n = 40 plasma) from COVID-19 patients infected during the first wave were tested by both the methods. ACE2 competition by each of the 10 variants present in the MSD-15 panel was individually compared with neutralizing antibody titers by PRNT_50_. The correlation between ACE2 competition and PRNT_50_ for every variant was determined by Spearman’s correlation. Spearman’s rho = r value varied from 0.76 to 0.83, whereas the p-value was <0.0001 for all the variants. **(B)** The MSD ACE2 assay was performed on plasma samples of COVID-19 patients naturally infected with the Wuhan virus (n = 19), B.1.617.1/Kappa lineage variant (n = 18), and B.1.617.2 strain/Delta lineage variant (n = 19). Individual variant-wise percent antibody positivity in the patient groups is depicted. **(C)** For the comparisons of anti-variant antibody levels in the three patient groups, ACE2 competition values of each variant were compared in all three patient groups (Wuhan, Kappa, Delta). Significance was assessed by Kruskal–Wallis test (*post- hoc* test– Dunn’s test). This analysis included all the patients irrespective of antibody positivity (n = 19 for Delta and Wuhan patients and n = 18 for Kappa patients). The value above each bar represents the median ACE2 competition value; variations measure the interquartile range (25%–75%). **(D)** The table includes significant p values obtained from *post- hoc* Dunn’s test after each variant’s comparison within all patient groups. The order used in the table nomenclature was the name of a particular variant followed by the name of the patient group (Wuhan, Kappa, Delta). **(E)** For this analysis, samples positive for anti-variant antibodies were considered for assessing the cross-reactivity of the generated antibodies. For the comparisons of anti-variant antibody levels in the three patient groups, ACE2 competition values were compared across the 10 variants in all three patient groups. ACE2 competition values of each variant were compared in all three patient groups (only antibody positive patient samples). Significance was assessed by the Kruskal–Wallis test (*post- hoc* test– Dunn’s test). This analysis includes antibody positive patient samples (Delta (n = 13–15) and Wuhan patients (n = 12–17) for Kappa patients (n = 5–8); sample number for each variant changes as only antibody- positive samples were taken. The value above each bar represents the median ACE2 competition value; variations measure the interquartile range (25%–75%). **(F)** The table includes significant p values obtained from *post- hoc* Dunn’s test after each variant’s comparison within all patient groups. The order used in the table nomenclature was the name of a particular variant followed by the name of the patient group (Wuhan, Kappa, Delta).The dotted line indicates the cutoff value (≥1.5) for ACE2 competition (fold inhibition). P values <0.05 were considered significant.

We first assessed the performance of the MSD assay. None of the 38 IgG-anti-SARS-CoV-2 negative pre-vaccination plasma samples scored reactive against all the 10 variants when a cutoff value of ≥1.5-fold was used in the MSD assay. Next, we compared the MSD assay with a live virus neutralization assay (PRNT_50_) that uses the Wuhan-Hu-1 strain (B.1, D614G-Wild-type). For this, seven serum and 40 plasma samples (n = 47) from the first wave of COVID-19 patients were used. ACE2 competition by the MSD assay and antibody titers from PRNT_50_ assay showed excellent correlation when all the variants used in the MSD assay were considered (r = 0.76–0.83, p < 0.0001, **Figure 1A**). Despite differences in the biologic properties of the variants, the cross reactivity of antibodies against multiple variants might explain why the PRNT shows high correlation with other variants in addition to Wuhan.

### 3.2 Variations in neutralization potential of COVID-19 patients infected with different SARS-CoV-2 variants

In India, the first COVID-19 wave was dominated by the Wuhan-Hu-1 strain (B.1), whereas the second wave was initially caused by the B.1.617.1/Kappa lineage variant and later by the B.1.617.2/Delta lineage variant (20). We first compared anti-variant-antibody seropositivity among the three patient groups (**Figure 1B**). Percent seropositivity against different variants was comparable among Wuhan (63%–89%) and Delta (68%–79%) variant-infected patients. However, the majority of Kappa-infected patients (56%–72%) were antibody negative. Although the number of patients was small, when the samples were collected within 1 week of the onset of symptoms, detectable anti-variant antibodies were not present in the majority of the Kappa patients.

To understand variant-specific quantitative differences in the patients infected with different variants, ACE2 competition was compared. The comparisons were performed for each variant among all three patient groups (Wuhan, Kappa, Delta). In accordance with the highest antibody negativity in the Kappa patients, the median ACE2 competition was lowest in this patient group across all the variants (**Figure 1C**). **Figure 1D** provides variant pairs with significant differences in the patient groups.

In view of the lack of antibodies in the majority of Kappa patients, we considered only antibody-positive samples (**Figure 1E**). This analysis revealed that whenever antibodies were present at the time of blood collection, both Kappa and Delta patients exhibited high and comparable neutralizing antibody levels against different variants. Majority significant differences in ACE2 competition were seen among Wuhan and Delta patient categories except for AY.1 and AY.2 variant in the Kappa patient category (**Figure 1F**). We would like to point out here that the Kappa variant has E484Q mutation. The 484 position in the RBD region is crucial for the interaction of neutralizing antibodies; mutation at this position resulted in a reduction in neutralizing antibody titer (20). In addition, E484Q mutation hinders electrostatic bonds at E484 and K31 in the Spike RBD region affecting interaction with ACE2 (23). Delta variants carry L452R and T478K (RBD region) mutations that affect binding at ACE2 by enhancing the stabilization of the ACE2–RBD complex (23). These mutations have been shown to alter viral interaction with ACE2 and might have affected antibody response in these patient groups. In the absence of follow-up samples, a subsequent comparison was not possible.

### 3.3 Neutralization potential against the major VOCs is reduced in COVISHIELD vaccine recipients

After understanding variant-specific ACE2 competition following natural infection, we evaluated the ability of anti-SARS-CoV-2 antibodies generated by the Wuhan strain-based COVISHIELD vaccine in targeting SARS-CoV-2 variants employed in the MSD assay. For this, vaccinees negative (prenegatives) and positive (prepositives) for IgG-anti-SARS-CoV-2 antibodies before immunization were analyzed separately. Of the prepositives, only three gave a history of prior COVID-19 (20–24 weeks before vaccination). The remaining vaccinees had the subclinical infection. To understand the durability of anti-variant antibodies, Nb levels were compared at 1 month when an optimum response is expected and at 6 months post-second dose (PD2-1, PD2-6, respectively). Two types of comparisons were made for both prenegatives and prepositives: (1) differences in the variant-specific Nab levels at any given point and (2) comparative decline of variant-specific neutralizing antibodies at 6 months post-vaccination.

### 3.4 Low anti-variant neutralizing antibody response in the prenegatives

**Figure 2:**
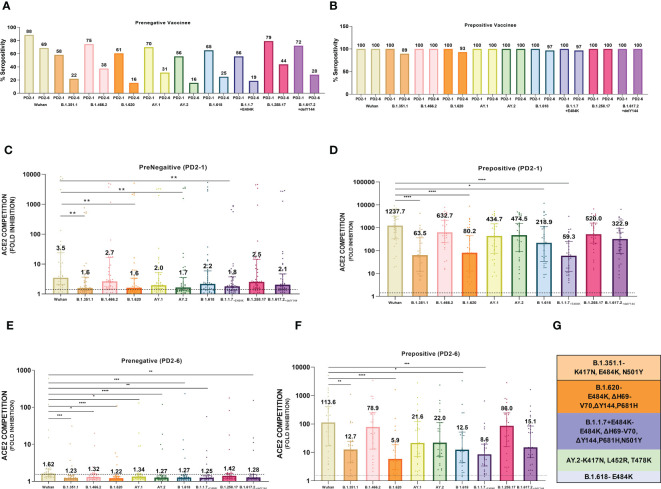
Plasma samples from COVISHIELD vaccine recipients either negative (prenegative) or positive (prepositive) for IgG-anti-SARS-CoV-2 antibodies were tested for anti-variant antibodies in the MSD-15 assay at 1 (PD2-1) and 6 months (PD2-6) after complete vaccination with two doses. The cutoff value for the assay was > 1.5. **(A, B)** provide variant-specific percent antibody positivity among prenegatives and prepositives respectively at both the time points. The value above each bar represents % seropositivity. ACE2 competition (fold inhibition) values were compared between all the variants at PD2-1 and PD2-6 among prenegatives **(C, E)** and prepositives **(D, F)**. Inter-variant comparisons were made for both the vaccinee groups at both the timepoints using the Kruskal–Wallis test (*post- hoc* test– Dunn’s test). The dotted line indicates the cutoff value (≥1.5). The value above each bar represents the median ACE2 competition value; variation measures the interquartile range (25%–75%), n = 43 PD2-1 prenegative vaccinee, n = 32 PD2-6 prenegative vaccinee, n = 32 PD2-1 prepositive vaccinee, n = 28 PD2-6 prepositive vaccinee. Stars expressing p values are: ****p < 0.0001, ***p < 0.001, **p < 0.01, and *p < 0.05. **(G)** denotes mutations identified in the respective variants with confirmed immune evasion by experimental studies. A similar color scheme was used to identify variants in the graph and corresponding mutations in the table.

At 1 month post-vaccination (**Figure 2A**), antibody positivity for the Wuhan strain was 88.4% in MSD, which was comparable (p = 1) to 95% (114/120) in PRNT_50_ when the same vaccinees were tested earlier (21). Surprisingly, almost 40% of the vaccinees lacked antibodies against AY.2, B.1.1.7+E484K, B.1.351.1, and B.1.620 (p = 0.002–0.006). These variants except AY.2 exhibit E484K mutation shown to be responsible for the reduction in neutralization by convalescent plasma from Wuhan-virus-infected patients and various monoclonal antibodies targeting receptor binding domain (**Figure 2G**) (24–26). AY.2 and B.1.351.1 variants possess K417N mutation, which is responsible for the reduction in ACE2 binding (**Figure 2G**) (27). Clearly, even when optimum antibody response is expected, a large proportion of the prenegative vaccinees were at risk of infection from these variants. For the other variants, positivity was comparable with Wuhan.

At 6 months, antibody positivity decreased for all the variants (**Figure 2A**). When compared with the Wuhan strain (68.8%), a lower proportion of antibody positives (15.6– 37.5%, p = 0.02- <0.001) were detected for eight variants; the difference with B.1.258.17 (43.8%) was not significant (p = 0.07). Thus, by 6 months post-immunization, the majority of the prenegatives lacked anti-variant neutralizing antibodies and could be susceptible to infections with a variety of variants.

Median ACE-2 competition was significantly reduced for B.1.351.1, B.1.620, AY.2, and B.1.1.7+E484K variants at 1 month post-vaccination when compared with the Wuhan virus (1.93–2.22- fold) (**Figure 2C**, p < 0.005 for all, **Table 1**). Antibody positivity and levels were lower against variants that are characterized by mutations that alter the antigenicity and immune evasion (**Table 1**). At 6 months, a significant decline in Nab levels was recorded for additional four variants. Except for Wuhan (median ± IQR, 1.6; 1.4–2.1), median ACE2 competition against all the other variants was below the cutoff value of 1.5 (**Figure 2E**). Taken together, Nab response to the variants known to have immune evasion-associated mutations was inferior even at 1 month and declined sharply for all the variants by 6 months.

### 3.5 Higher and durable anti-variant Nab response in the prepositives

At 1 month post-immunization, all the 32 vaccinees with hybrid immunity were Nab positive for all the variants examined (**Figure 2B**). As the vaccine was administered post- first wave, the highest ACE2 competition was observed against the Wuhan strain (**Figure 2D**). High fold reduction in ACE-2 binding (5.6–20.9-fold, p = 0.039–<0.0001) was recorded for B.1.351.1, B.1.620, B.1.618, and B.1.1.7+E484K variants with characteristic mutations in the S protein (**Table 1**; **Figure 2D**). Notably, except B.1.618, these variants are classified by the WHO as VOCs. At the 6- month follow-up, a small proportion (3.5%–10.7%, **Figure 2B**) of the prepositive vaccinees circulating lower Nab levels at 1 month turned out antibody- negative against the same four variants. Nab levels continued to be lower for these variants, whereas comparable levels were recorded against the remaining variants (**Figure 2F**). Overall, irrespective of the prior antibody positivity, Nab levels were consistently lower for the VOCs included in the MSD panel.

**Figure 3:**
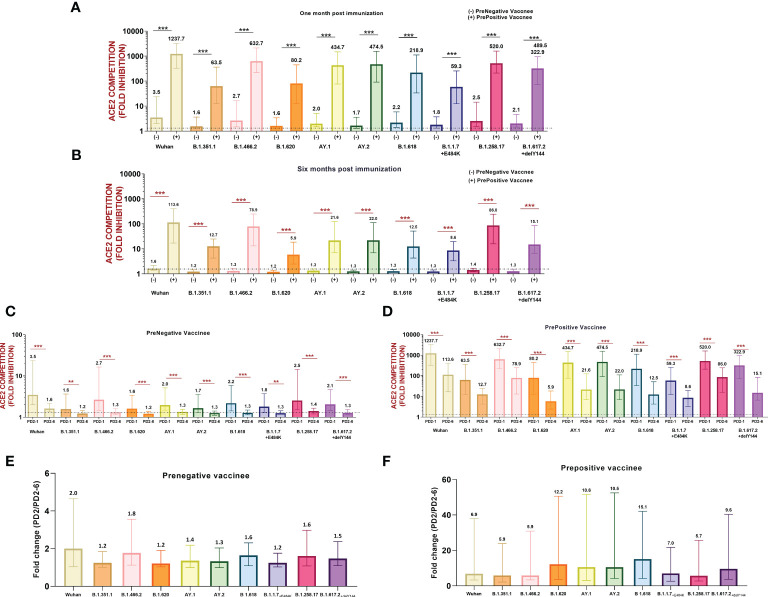
**(A)** Variant-wise comparison of ACE2 competition between prenegative (-) and prepositive (+) vaccinees **(A)** at 1 month and **(B)** 6 months post-immunization. ACE2 competition of each variant in both the groups was compared by the Mann–Whitney test (***p < 0.001, **p < 0.01). Neutralizing antibody levels/ACE2 competition values of each variant were compared at PD2-1 and PD2-6 in **(C)** prenegative and **(D)** prepositive vaccinees. Statistical significance was assessed by the Mann–Whitney U test. ***p < 0.001, **p < 0.01. In **(A-D)**, the value above each bar represents the median ACE2 competition value; variations measure the interquartile range (25%–75%), n = 43 for PD2-1 prenegative vaccinees, n = 32 for PD2-6 prenegative vaccinees, n = 32 for PD2-1 prepositive vaccinees, and n = 28 for PD2-6 prepositive vaccinees. **(E, F)** respectively depict fold changes in neutralizing antibody levels at PD2-1 and PD2-6 (PD2-1/PD2-6) for each variant among prenegatives (n = 32 pairs) and prepositives (n = 28 pairs). Inter-variant comparisons were performed by one-way ANOVA. In **(E, F)**, the value above each bar represents median fold change (PD2-1/PD2-6); variations measure the interquartile range (25%–75%).

Next, we compared variant-specific Nab responses among prenegative and prepositive vaccinees at PD2-1 and PD2-6 (**Figures 3A, B**). At both time points, vaccinees with hybrid immunity exhibited remarkably higher antibody levels against all the 10 variants (p ≤ 0.0001). However, irrespective of prior antibody positivity and levels, a significant decline was recorded for all the variants at 6 months (p ≤ 0.001–<0.0001, **Figures 3C, D**) in both groups. To understand the variant-specific decline in the vaccinees, fold changes in Nab levels at 1 and 6 months were compared (**Figures 3E, F**). The degree of decline was independent of the variants suggesting that antibodies against different variants decline at comparable levels at least for 6 months post-immunization.

## 4 Discussion

In this study, we demonstrated that anti-variant neutralizing antibody responses of non-vaccinated COVID-19 patients infected with Kappa or Delta variants and the parenteral B.1 strain differ strikingly and to a variable extent. Our data suggest that the anti-variant immune response to vaccines will be influenced by the type of the variant infecting either pre- or post-vaccination/booster dose. Furthermore, our previous observations of superior immune response against the B.1 strain among prepositive than prenegative recipients of COVISHIELD vaccine even at 6 months vaccination (21) are extended to the other non-Omicron VOCs. Although higher, Nab levels against the Alpha, Beta, and Delta variants were compromised even in prepositives. Most of the prenegative vaccinees might be infected with a variety of variants at 1 month post-vaccination, almost all being susceptible at 6 months.

We selected MSD-ACE2 panel-15 available at the time of conducting the study and found an excellent correlation with PRNT_50_. At that time, the Omicron variant was not yet added. Employing an earlier MSD panel-7 with RBD from wild type and B.1.351(Beta), B.1.1.7(Alpha), and P.1(Gamma) variants, Pegu et al. observed comparable antibody responses against mRNA-1273 vaccine when a live virus/pseudovirus neutralization and MSD assays were performed (28). An elegant study by Sammartino et al. assessing the immunogenicity of the BNT162b2 vaccine deserves special mention (19). The investigators isolated, characterized, and used 14 SARS-CoV-2 variants in MNT using a 90% reduction in CPE as the indicator. This requires extraordinary efforts, resources, and time. Although only 5/14 strains used here are similar to the one used in the MSD panel, it is noteworthy that the results obtained by MNT and MSD are comparable when vaccines based on wild-type strains are evaluated (19). The requirement of expensive equipment remains a significant limitation for the use of this platform in laboratories with limited resources. However, once the machine is available, the cost of the test kits can be compensated by the ability of the assay to determine the presence and levels of neutralizing antibodies against multiple variants in just 2.5 h. So far, studies on the cross-reactivity of neutralizing antibodies in patients infected with emerging variants are limited (17, 29). We found that in the patients infected with the Kappa variant, antibody positivity was low. However, the observed differences were not related to the time of sample collection as median PODs were not different for the patients infected with different variants (data not shown). In the patients infected during the first wave, we previously showed that Nab positivity (PRNT_50_) during the first week post-onset of clinical symptoms was 61.5% that rose to 95.4% during the second week with concomitant rise in Nab titers. In the absence of the Kappa variant in the panel, whether Nab reactivity/titers against the homologous virus was higher could not be ascertained.

Among antibody- positive Kappa patients, the anti-variant antibody levels were comparable with Delta patients. The common mutations of significance (L452R and P681R) present in both the variants may have been responsible for similar antibody reactivity patterns with the other variants. On phylogenetic analysis, these variants were shown to form separate branches of the same cluster (30, 31). On the other hand, the Wuhan virus (B.1) eliciting lower antibody levels was distinctly different. Similarly, in accordance with the specific mutations altering biological characteristics, the other VOCs belonged to distinct clusters. By using a live virus focus reduction neutralization test, Edara et al. (32) showed that convalescent sera from B.1.617 (Kappa variant)- infected patients exhibited a 6.5 times reduction in neutralization than with the WA1/2020 (Wuhan) virus. Furthermore, by performing mutation- specific pseudovirus-based study, Yang et al. identified that the E484Q mutation (present in the Kappa variant as well) in the spike region of the B.1.617.1 variant is the major contributor to the reduction in neutralization (33, 34).

While comparing antibody cross-reactivity in COVID-19 patients admitted to the ICU during the first (Wild type) and third (Alpha/mixed Wild type) waves employing the multiplex Luminex-based assay, Fraser et al. observed that wave-3 patients developed a robust neutralizing antibody response to Wild-type, Alpha, Delta, and to a lesser extent to Beta and Gamma VOCs (35). A similar neutralizing antibody response was observed in the current study. Cross-reactivity of Delta patients inducing high antibody titers was significantly less against the Beta variant and also with the Alpha variant (Gamma VOC was not present in the MSD panel). Taken together, both Alpha (Fraser et al.) and Delta (current study) virus-infected patients seem to be better antibody producers. Another possibility is subclinical infection during the first wave (at least in a few) with subsequent exponential antibody rise following reinfection during the subsequent waves. However, higher antibody negativity in the Kappa patients studied during the same time argues against this possibility. Both of the studies did not elicit a history of COVID-19. Whenever vaccines will be administered to individuals infected with such variants, the resultant antibody response could be distinctly different than vaccinees infected earlier with the Wuhan virus. For variants emerging in the future, it will be difficult to assess immune response in antibody- naïve individuals as the majority of the population will either be vaccinated or infected or vaccinated and infected with clinical or subclinical presentations.

COVID-19 vaccine efficacy is being tested against multiple emerging variants with capabilities to evade vaccine-induced antibodies. COVISHIED is the major vaccine used in India and hence will have a major impact on the success of overall vaccination. ~40% Nab negativity against major VOCs at PD2-1 when an optimum response is expected and ~100% negativity at 6 months indeed is a matter of great concern. This did result in a large number of breakthrough infections with Delta and Omicron VOCs (36–38). During the explosive Delta wave, the efficacy of the COVISHIELD vaccine against clinical disease was shown to be 61.3% in Chennai, India (39). In view of such observations, a booster dose termed as “precautionary dose” was introduced in India and an additional dose is now being recommended. As of 01/03/2023, 2.2 billion COVID-19 vaccine doses have been administered, which includes 227.1 million recipients of the precautionary dose (cowin.gov.in).

We, along with others, have shown remarkably superior immune responses in vaccinees with hybrid immunity (16, 21). The immune response to a vaccine varies with populations, prior antibody positivity, and the type of antibody tests used. We compared the immune response of the COVISHIELD vaccine used in India with AZD-1222, the early counterpart of COVISHIELD vaccine, administered in Germany (40), Taiwan (41) Africa (42), and Thailand (43). The tests used were pseudotype neutralization for the first three and an ELISA-based surrogate neutralization test for the fourth study. The overall neutralization results obtained by administering either COVISHIELD (India) or AZD-1222 (four countries mentioned above) were comparable. In some countries, different vaccines were used depending on their availability. In Taiwan (41) and Thailand (43), mRNA vaccines performed better than AZD-1222 while though mRNA vaccines elicited higher Nab levels in Germany (40), fold reduction was high for the Beta variant and similar for the Delta variant. A two-dose regimen of the AZD1222 vaccine in South Africa did result in a lower antibody response using a live B.1.351 variant neutralization assay, and the vaccine did not show protection against mild-to-moderate COVID-19 (44). In another study, lower Nab titers were recorded against B.1.1.7, B.1.351, and B.1.617.2 using a live virus neutralization assay (45).

In India, COVISHIELD and COVAXIN (adjuvanted, whole virus inactivated) are the major vaccines used for primary immunization. Several studies documented higher antibody response by COVISHIELD than COVAXIN (46–48) Our data showed that COVISHIELD was a better neutralizing antibody producer whereas Covaxin induced higher T- cell responses (21). However, studies on the response to variants are limited (49, 50). The present study attempts to provide this useful information.

The current study has some limitations. In the absence of the Omicron variant in the panel available at the time of conducting the study, we are not able to provide crucial information about this currently circulating global variant. Convalescent samples from patients were not available and hence the differences in neutralization activity at the height of antibody response could not be determined. Nonetheless, using small volumes of available samples, we were able to provide the required information on variant-specific Nab response in patients infected with different variants and COVISHIELD vaccine recipients. Detailed analysis of immune response among breakthrough infections with different variants will provide some answers to the relationship of vaccine-induced variant-dependent, and infection-induced immune responses. In view of the comparable results obtained by PRNT_50_ and MSD assay and the convenience and ease of a single assay, the MSD platform could be an attractive multiplex alternative for the assessment of neutralizing antibodies to multiple variants.

## Data availability statement

The original contributions presented in the study are included in the article/Supplementary Material. Further inquiries can be directed to the corresponding author.

## Ethics statement

The studies involving human participants were reviewed and approved by institutional “Human Ethics Committee” (No: BVDUMC/IEC/185A). The patients/participants provided their written informed consent to participate in this study.

## Author contributions

RajP performed MSD experiments and related analysis and wrote the manuscript. SP was responsible for the recruitment of patients/vaccinees and collection of the relevant information/blood samples. AM analyzed the data and reviewed the manuscript. VA conceived and designed the study, supervised the work and data analysis, and critically reviewed the manuscript. RahP has performed statistical analysis. All authors contributed to the article and approved the submitted version.

## References

[1] Hou YJ Chiba S Halfmann P Ehre C Kuroda M Dinnon KH. SARS-CoV-2 D614G variant exhibits efficient replication ex vivo and transmission *in vivo* . Science (2020) 370:1464–8. doi: 10.1126/SCIENCE.ABE8499 PMC7775736 33184236

[2] Korber B Fischer WM Gnanakaran S Yoon H Theiler J Abfalterer W. Tracking changes in SARS-CoV-2 spike: evidence that D614G increases infectivity of the COVID-19 virus. Cell (2020) 182:812–827.e19. doi: 10.1016/J.CELL.2020.06.043 32697968 PMC7332439

[3] Preliminary genomic characterisation of an emergent SARS-CoV-2 lineage in the UK defined by a novel set of spike mutations - SARS-CoV-2 coronavirus/nCoV-2019 genomic epidemiology - virological. Available at: https://virological.org/t/preliminary-genomic-characterisation-of-an-emergent-sars-cov-2-lineage-in-the-uk-defined-by-a-novel-set-of-spike-mutations/563/1 (Accessed February 16, 2023).

[4] Tegally H Wilkinson E Giovanetti M Iranzadeh A Fonseca V Giandhari J. Detection of a SARS-CoV-2 variant of concern in south Africa. Nature (2021) 592(7854):438–43. doi: 10.1038/s41586-021-03402-9 33690265

[5] Phylogenetic relationship of SARS-CoV-2 sequences from Amazonas with emerging Brazilian variants harboring mutations E484K and N501Y in the spike protein - SARS-CoV-2 coronavirus/nCoV-2019 genomic epidemiology - virological. Available at: https://virological.org/t/phylogenetic-relationship-of-sars-cov-2-sequences-from-amazonas-with-emerging-brazilian-variants-harboring-mutations-e484k-and-n501y-in-the-spike-protein/585 (Accessed February 16, 2023).

[6] Campbell F Archer B Laurenson-Schafer H Jinnai Y Konings F Batra N. Rapid communication increased transmissibility and global spread of SARS-CoV-2 variants of concern as at June 2021.1. (United kingdom, Switzerland: Eurosurveillance) (2021). doi: 10.2807/1560-7917.ES.2021.26.24.2100509. PMC8212592 34142653

[7] Viana R Moyo S Amoako DG Tegally H Scheepers C Althaus CL. Rapid epidemic expansion of the SARS-CoV-2 omicron variant in southern Africa. Nature (2022) 603:679. doi: 10.1038/S41586-022-04411-Y 35042229 PMC8942855

[8] Tegally H Moir M Everatt J Giovanetti M Scheepers C Wilkinson E. Emergence of SARS-CoV-2 omicron lineages BA.4 and BA.5 in south Africa. Nat Med (2022) 28(9):1785–90. doi: 10.1038/s41591-022-01911-2 PMC9499863 35760080

[9] Ba A Changed VOI Added VOC Ba A Replaced O. Changes to list of SARS-CoV-2 variants of concern, variants of interest, and variants under monitoring. Eur Centre Dis Prev Control (2022).

[10] Ortega MA García-Montero C Fraile-Martinez O Colet P Baizhaxynova A Mukhtarova K. Recapping the features of SARS-CoV-2 and its main variants: status and future paths. J Pers Med (2022) 12(6):995. doi: 10.3390/jpm12060995 35743779 PMC9225183

[11] Jha N Hall D Kanakan A Mehta P Maurya R Mir Q. Geographical landscape and transmission dynamics of SARS-CoV-2 variants across India: a longitudinal perspective. Front Genet (2021) 12:753648/BIBTEX. doi: 10.3389/FGENE.2021.753648/BIBTEX 34976008 PMC8719586

[12] Emerging variants of SARS-CoV-2 and novel therapeutics against coronavirus (COVID-19) . PubMed. (Accessed February 26, 2023). 34033342

[13] Scheepers C Everatt J Amoako DG Tegally H Wibmer CK Mnguni A. Emergence and phenotypic characterization of the global SARS-CoV-2 C.1.2 lineage. Nat Commun (2022) 13(1):1–9. doi: 10.1038/s41467-022-29579-9 35396511 PMC8993834

[14] Zhang L Cui Z Li Q Wang B Yu Y Wu J. Ten emerging SARS-CoV-2 spike variants exhibit variable infectivity, animal tropism, and antibody neutralization emerging mutations in SARS-CoV-2 cause several waves of COVID-19 pandemic. here we investigate the infectivity and antigenicity of ten emerging SARS-CoV-2 variants-b. China: Nature- Communications Biology (2021). pp. 526–7. doi: 10.1038/s42003-021-02728-4. PMC8514557 34645933

[15] Khoury DS Cromer D Reynaldi A Schlub TE Wheatley AK Juno JA. Neutralizing antibody levels are highly predictive of immune protection from symptomatic SARS-CoV-2 infection. Nat Med (2021) 27:1205–11. doi: 10.1038/S41591-021-01377-8 34002089

[16] Al-Sadeq DW Shurrab FM Ismail A Amanullah FH Thomas S Aldewik N. Comparison of antibody immune responses between BNT162b2 and mRNA-1273 SARS-CoV-2 vaccines in naïve and previously infected individuals. J Travel Med (2021) 28(8):taab190. doi: 10.1093/jtm/taab190 34888670 PMC8754698

[17] Stamatatos L Czartoski J Wan YH Homad LJ Rubin V Glantz H. mRNA vaccination boosts cross-variant neutralizing antibodies elicited by SARS-CoV-2 infection. Science (2021) 372:1413–8. doi: 10.1126/SCIENCE.ABG9175 PMC8139425 33766944

[18] Wall EC Wu M Harvey R Kelly G Warchal S Sawyer C. AZD1222-induced neutralising antibody activity against SARS-CoV-2 delta VOC. Lancet (2021) 398:207–9. doi: 10.1016/S0140-6736(21)01462-8 PMC8238446 34197809

[19] Sammartino JC Cassaniti I Ferrari A Giardina F Ferrari G Zavaglio F. Evaluation of the neutralizing antibodies response against 14 SARS-CoV-2 variants in BNT162b2 vaccinated naïve and COVID-19 positive healthcare workers from a northern Italian hospital. Vaccines (Basel) (2022) 10:703. doi: 10.3390/VACCINES10050703/S1 35632457 PMC9145000

[20] Shrivastava S Mhaske ST Modak MS Virkar RG Pisal SS Mishra AC. Emergence of two distinct variants of SARS-CoV-2 and an explosive second wave of COVID-19: the experience of a tertiary care hospital in pune, India. Arch Virol (2022) 167:393–403. doi: 10.1007/S00705-021-05320-7 35000004 PMC8742689

[21] Arankalle V Kulkarni-Munje A Kulkarni R Palkar S Patil R Oswal J. Immunogenicity of two COVID-19 vaccines used in India: an observational cohort study in health care workers from a tertiary care hospital. Front Immunol (2022) 13:928501. doi: 10.3389/FIMMU.2022.928501 36211366 PMC9540493

[22] Shrivastava S Palkar S Shah J Rane P Lalwani S Mishra AC. Early and high SARS-CoV-2 neutralizing antibodies are associated with severity in COVID-19 patients from India. Am J Trop Med Hyg (2021) 105:401–6. doi: 10.4269/AJTMH.21-0014 PMC8437163 34138748

[23] Cherian S Potdar V Jadhav S Yadav P Gupta N Das M. Sars-cov-2 spike mutations, l452r, t478k, e484q and p681r, in the second wave of covid-19 in maharashtra, India. Microorganisms (2021) 9:1542. doi: 10.3390/MICROORGANISMS9071542/S1 34361977 PMC8307577

[24] Starr TN Greaney AJ Dingens AS Bloom JD. Complete map of SARS-CoV-2 RBD mutations that escape the monoclonal antibody LY-CoV555 and its cocktail with LY-CoV016. Cell Rep Med (2021) 2(4):100255. doi: 10.1016/j.xcrm.2021.100255 33842902 PMC8020059

[25] Zhou D Dejnirattisai W Supasa P Liu C Mentzer AJ Ginn HM. Evidence of escape of SARS-CoV-2 variant B.1.351 from natural and vaccine-induced sera. Cell (2021) 184:2348–2361.e6. doi: 10.1016/J.CELL.2021.02.037 33730597 PMC7901269

[26] Cele S Gazy I Jackson L Hwa SH Tegally H Lustig G. Escape of SARS-CoV-2 501Y.V2 from neutralization by convalescent plasma. Nature (2021) 593:142–6. doi: 10.1038/S41586-021-03471-W PMC9867906 33780970

[27] Jawad B Adhikari P Podgornik R Ching WY. Key interacting residues between RBD of SARS-CoV-2 and ACE2 receptor: combination of molecular dynamics simulation and density functional calculation. J Chem Inf Model (2021) 61:4425–41. doi: 10.1021/ACS.JCIM.1C00560/ASSET/IMAGES/LARGE/CI1C00560_0010.JPEG 34428371

[28] Pegu A O’Connell SE Schmidt SD O’Dell S Talana CA Lai L. Durability of mRNA-1273 vaccine-induced antibodies against SARS-CoV-2 variants. Sci (1979) (2021) 373:1372–7. doi: 10.1126/SCIENCE.ABJ4176/SUPPL_FILE/SCIENCE.ABJ4176_MDAR_REPRODUCIBILITY_CHECKLIST.PDF PMC8691522 34385356

[29] Moyo-Gwete T Madzivhandila M Makhado Z Ayres F Mhlanga D Oosthuysen B. Cross-reactive neutralizing antibody responses elicited by SARS-CoV-2 501Y.V2 (B.1.351). N Engl J Med (2021) 384:2161–3. doi: 10.1056/NEJMC2104192 PMC8063886 33826816

[30] Sun Y Lin W Dong W Xu J. Origin and evolutionary analysis of the SARS-CoV-2 omicron variant. J Biosaf Biosecur (2022) 4:33–7. doi: 10.1016/J.JOBB.2021.12.001 PMC8718870 35005525

[31] Carabelli AM Peacock TP Thorne LG Harvey WT Hughes J de Silva TI. SARS-CoV-2 variant biology: immune escape, transmission and fitness. Nat Rev Microbiol (2023) 21(3):162–77. doi: 10.1038/s41579-022-00841-7 PMC9847462 36653446

[32] Edara V-V Pinsky BA Suthar MS Lai L Davis-Gardner ME Floyd K. Infection and vaccine-induced neutralizing-antibody responses to the SARS-CoV-2 B.1.617 variants. New Engl J Med (2021) 385:664–6. doi: 10.1056/NEJMC2107799/SUPPL_FILE/NEJMC2107799_DISCLOSURES.PDF PMC8279090 34233096

[33] Yang X Zhu Y Xun J Liu J Wen Q Lin Y. The neutralization of B.1.617.1 and B.1.1.529 sera from convalescent patients and BBIBP-CorV vaccines. iScience (2022) 25(9):105016. doi: 10.1016/j.isci.2022.105016 36062074 PMC9420027

[34] Hoffmann M Hofmann-Winkler H Krüger N Kempf A Nehlmeier I Graichen L. SARS-CoV-2 variant B.1.617 is resistant to bamlanivimab and evades antibodies induced by infection and vaccination. Cell Rep (2021) 36(3):109415. doi: 10.1016/j.celrep.2021.109415 34270919 PMC8238662

[35] Fraser DD Patel MA van Nynatten LR Martin C Seney SL Miller MR. Cross-immunity against SARS-COV-2 variants of concern in naturally infected critically ill COVID-19 patients. Heliyon (2023) 9(1):e12704. doi: 10.1016/j.heliyon.2022.e12704 36594041 PMC9797417

[36] Malhotra S Mani K Lodha R Bakhshi S Mathur VP Gupta P. COVID-19 infection, and reinfection, and vaccine effectiveness against symptomatic infection among health care workers in the setting of omicron variant transmission in new Delhi, India. Lancet Regional Health - Southeast Asia (2022) 3:100023. doi: 10.1016/j.lansea.2022.100023 35769163 PMC9167830

[37] Kale P Gupta E Bihari C Patel N Rooge S Pandey A. Vaccine breakthrough infections by SARS-CoV-2 variants after ChAdOx1 nCoV-19 vaccination in healthcare workers. Vaccines (Basel) (2022) 10(1):54. doi: 10.3390/vaccines10010054 PMC8778656 35062715

[38] Gerges D Kapps S Hernández-Carralero E Freire R Aiad M Schmidt S. Vaccination with BNT162b2 and ChAdOx1 nCoV-19 induces cross-reactive anti-RBD IgG against SARS-CoV-2 variants including omicron. Viruses (2022) 14(6):1181. doi: 10.3390/v14061181 35746653 PMC9231407

[39] Murali S Sakthivel M Pattabi K Venkatasamy V Thangaraj JWV Shete A. Effectiveness of the ChAdOx1 nCoV-19 coronavirus vaccine (CovishieldTM) in preventing SARS-CoV2 infection, chennai, Tamil nadu, India, 2021. Vaccines (Basel) (2022) 10(6):970. doi: 10.3390/vaccines10060970 35746578 PMC9228854

[40] Jacobsen H Strengert M Maaß H Ynga Durand MA Katzmarzyk M Kessel B. Diminished neutralization responses towards SARS-CoV-2 omicron VoC after mRNA or vector-based COVID-19 vaccinations. Sci Rep (2022) 12:19858. doi: 10.1038/s41598-022-22552-y 36400804 PMC9673895

[41] Chao CH Cheng D Huang SW Chuang YC Yeh TM Wang JR. Serological responses triggered by different SARS-CoV-2 vaccines against SARS-CoV-2 variants in Taiwan. Front Immunol (2022) 13:1023943/BIBTEX. doi: 10.3389/FIMMU.2022.1023943/BIBTEX 36458016 PMC9705976

[42] Madhi SA Kwatra G Richardson SI Koen AL Baillie V Cutland CL. Durability of ChAdOx1 nCoV-19 (AZD1222) vaccine and hybrid humoral immunity against variants including omicron BA.1 and BA.4 6 months after vaccination (COV005): a post-hoc analysis of a randomised, phase 1b-2a trial. Lancet Infect Dis (2023) 23:295–306. doi: 10.1016/S1473-3099(22)00596-5 36273491 PMC9584570

[43] Sudjaritruk T Mueangmo O Saheng J Winichakoon P Salee P Wongjak W. Comparison of immunogenicity and reactogenicity of five primary series of COVID-19 vaccine regimens against circulating SARS-CoV-2 variants of concern among healthy Thai populations. Vaccines (Basel) (2023) 11:564. doi: 10.3390/VACCINES11030564 36992147 PMC10051392

[44] Madhi SA Baillie V Cutland CL Voysey M Koen AL Fairlie L. Efficacy of the ChAdOx1 nCoV-19 covid-19 vaccine against the B.1.351 variant. N Engl J Med (2021) 384:1885–98. doi: 10.1056/NEJMOA2102214 PMC7993410 33725432

[45] Wall EC Wu M Harvey R Kelly G Warchal S Sawyer C. Neutralising antibody activity against SARS-CoV-2 VOCs B.1.617.2 and B.1.351 by BNT162b2 vaccination. Lancet (2021) 397:2331–3. doi: 10.1016/S0140-6736(21)01290-3 PMC8175044 34090624

[46] Satpathi PS Endow S Gantait K Chand PH Jaiswal A Jana B. Anti SARS-CoV 2 IgG antibody response in fully vaccinated covishield (AZD1222) and covaxin (BBV-152) recipients: a study done in southern part of West Bengal, India. Iran J Microbiol (2022) 14:611–6. doi: 10.18502/IJM.V14I5.10953 PMC9723424 36531814

[47] Mog C Bhattacharya S Baidya S Das S. Antibody responses of SARS-CoV-2 vaccines amongst health care workers in a tertiary care hospital in tripura, India: a cross-sectional study. Indian J Community Med (2022) 47:583–6. doi: 10.4103/IJCM.IJCM_71_22 PMC9891050 36742975

[48] Singh AK Phatak SR Singh R Bhattacharjee K Singh NK Gupta A. Humoral antibody kinetics with ChAdOx1-nCOV (Covishield™) and BBV-152 (Covaxin™) vaccine among Indian healthcare workers: a 6-month longitudinal cross-sectional coronavirus vaccine-induced antibody titre (COVAT) study. Diabetes Metab Syndr (2022) 16(2):102424. doi: 10.1016/j.dsx.2022.102424 35150961 PMC8816907

[49] Yadav PD Sapkal GN Abraham P Deshpande G Nyayanit DA Patil DY. Neutralization potential of covishield vaccinated individuals sera against B.1.617.1. Clin Infect Dis (2022) 74:558–9. doi: 10.1093/CID/CIAB483 34036309

[50] Sapkal G Kant R Dwivedi G Sahay RR Yadav PD Deshpande GR. Immune responses against different variants of SARS-CoV-2 including omicron following 6 months of administration of heterologous prime-boost COVID-19 vaccine. J Travel Med (2022) 29(3):taac033. doi: 10.1093/jtm/taac033 35244698 PMC8903478

